# Supplementary material for: Spatial and temporal clustering of anti-SARS-CoV-2 antibodies in Illinois household cats, 2021–2023
Source: PLoS One. 2024 May 2;19(5):e0299388. doi: 10.1371/journal.pone.0299388 (PMC11065222; doi:10.1371/journal.pone.0299388)
Supplement: S1 Table — (DOCX) [file pone.0299388.s001.docx]

**S1 Table. ELISA and Lumit™ Dx SARS-CoV-2 Immunoassay results for cat serum samples.** bELISA cut off value = 17.6%; Lumit assay cut off value = 1.

| **Number** | **Sample ID** | **ELISA PI (%)** | **Lumit** | **Status** |
| --- | --- | --- | --- | --- |
| 1 | Sample#9 | 77.56 | 1.71 | Positive |
| 2 | Sample#46 | 21.17 | 2.58 | Positive |
| 3 | Sample#71 | 20.11 | 1.98 | Positive |
| 4 | Sample#84 | 60.93 | 1.30 | Positive |
| 5 | Sample#86 | 28.06 | 2.67 | Positive |
| 6 | Sample#91 | 27.51 | 15.25 | Positive |
| 7 | Sample#93 | 20.40 | 1.27 | Positive |
| 8 | Sample#94 | 79.69 | 1.28 | Positive |
| 9 | Sample#112 | 95.00 | 2.01 | Positive |
| 10 | Sample#123 | 19.22 | 1.23 | Positive |
| 11 | Sample#161 | 100.06 | 798.27 | Positive |
| 12 | Sample#166 | 17.99 | 1.24 | Positive |
| 13 | Sample#167 | 23.40 | 1.40 | Positive |
| 14 | Sample#169 | 64.19 | 1.24 | Positive |
| 15 | Sample#173 | 45.22 | 2.44 | Positive |
| 16 | Sample#174 | 22.67 | 2.64 | Positive |
| 17 | Sample#182 | 30.68 | 2.10 | Positive |
| 18 | Sample#183 | 24.51 | 2.55 | Positive |
| 19 | Sample#184 | 19.12 | 4.90 | Positive |
| 20 | Sample#187 | 22.41 | 4.23 | Positive |
| 21 | Sample#195 | 47.20 | 3.01 | Positive |
| 22 | Sample#197 | 25.00 | 1.59 | Positive |
| 23 | Sample#211 | 41.30 | 1.32 | Positive |
| 24 | Sample#216 | 24.73 | 613.73 | Positive |
| 25 | Sample#217 | 99.98 | 40.29 | Positive |
| 26 | Sample#220 | 17.94 | 9.82 | Positive |
| 27 | Sample#255 | 18.83 | 1.54 | Positive |
| 28 | Sample#256 | 19.16 | 1.56 | Positive |
| 29 | Sample#274 | 22.92 | 3.68 | Positive |
| 30 | Sample#290 | 100.04 | 7.76 | Positive |
| 31 | Sample#528 | 22.59 | 1.96 | Positive |
| 32 | Sample#542 | 33.21 | 5.30 | Positive |
| 33 | Sample#563 | 17.72 | 9.34 | Positive |
| 34 | Sample#567 | 19.33 | 6.92 | Positive |
| 35 | Sample#581 | 87.47 | 175.73 | Positive |
| 36 | Sample#587 | 23.15 | 6.92 | Positive |
| 37 | Sample#607 | 24.62 | 8.37 | Positive |
| 38 | Sample#624 | 23.80 | 1.65 | Positive |
| 39 | Sample#639 | 61.82 | 300.96 | Positive |
| 40 | Sample#648 | 23.03 | 319.05 | Positive |
| 41 | Sample#651 | 31.45 | 4.32 | Positive |
| 42 | Sample#655 | 24.77 | 642.62 | Positive |
| 43 | Sample#662 | 21.79 | 355.69 | Positive |
| 44 | Sample#674 | 26.20 | 1.15 | Positive |
| 45 | Sample#685 | 79.23 | 166.57 | Positive |
| 46 | Sample#711 | 18.93 | 6.27 | Positive |
| 47 | Sample#762 | 23.86 | 362.60 | Positive |
| 48 | Sample#866 | 30.72 | 1.83 | Positive |
| 49 | Sample#874 | 33.28 | 149.38 | Positive |
| 50 | Sample#888 | 55.35 | 190.53 | Positive |
| 51 | Sample#892 | 52.95 | 246.34 | Positive |
| 52 | Sample#908 | 24.19 | 336.53 | Positive |
| 53 | Sample#909 | 19.86 | 269.73 | Positive |
| 54 | Sample#941 | 23.25 | 1.20 | Positive |
| 55 | Sample#952 | 36.21 | 271.70 | Positive |
| 56 | Sample#954 | 99.38 | 807.12 | Positive |
| 57 | Sample#965 | 76.66 | 558.20 | Positive |
| 58 | Sample#999 | 25.79 | 204.48 | Positive |
| 59 | Sample#1023 | 17.68 | 175.45 | Positive |
| 60 | Sample#1030 | 31.43 | 115.83 | Positive |
| 61 | Sample#1048 | 42.26 | 1.18 | Positive |
| 62 | Sample#1053 | 54.49 | 168.83 | Positive |
| 63 | Sample#1060 | 47.26 | 461.87 | Positive |
| 64 | Sample#1068 | 96.79 | 1.41 | Positive |
| 65 | Sample#1073 | 21.81 | 1.22 | Positive |
| 66 | Sample#1081 | 35.25 | 486.19 | Positive |
| 67 | Sample#1084 | 38.73 | 90.12 | Positive |
| 68 | Sample#1088 | 70.00 | 64.42 | Positive |
| 69 | Sample#1092 | 85.80 | 155.72 | Positive |
| 70 | Sample#1098 | 70.46 | 1.95 | Positive |
| 71 | Sample#1099 | 21.20 | 1.09 | Positive |
| 72 | Sample#1110 | 45.55 | 1.03 | Positive |
| 73 | Sample#1114 | 25.24 | 2.78 | Positive |
| 74 | Sample#1135 | 42.44 | 1.27 | Positive |
| 75 | Sample#1150 | 40.12 | 1.49 | Positive |
| 76 | Sample#1189 | 25.15 | 1.49 | Positive |
| 77 | Sample#1192 | 17.90 | 1.51 | Positive |
| 78 | Sample#1200 | 34.74 | 639.94 | Positive |
| 79 | Sample#1222 | 22.64 | 1.40 | Positive |
| 80 | Sample#1223 | 23.09 | 1.24 | Positive |
| 81 | Sample#1225 | 35.94 | 2.44 | Positive |
| 82 | Sample#1235 | 35.15 | 2.64 | Positive |
| 83 | Sample#1243 | 22.71 | 1228.69 | Positive |
| 84 | Sample#1245 | 24.52 | 7.13 | Positive |
| 85 | Sample#1249 | 21.15 | 426.50 | Positive |
| 86 | Sample#1252 | 72.61 | 11.79 | Positive |
| 87 | Sample#1265 | 26.20 | 39.32 | Positive |
| 88 | Sample#1269 | 20.59 | 79.03 | Positive |
| 89 | Sample#1274 | 25.53 | 1005.79 | Positive |
| 90 | Sample#1276 | 83.28 | 2.48 | Positive |
| 91 | Sample#1277 | 22.97 | 2.42 | Positive |
| 92 | Sample#1281 | 30.13 | 2.85 | Positive |
| 93 | Sample#1282 | 24.35 | 1178.19 | Positive |
| 94 | Sample#1284 | 26.78 | 2.22 | Positive |
| 95 | Sample#1290 | 42.77 | 1206.01 | Positive |
| 96 | Sample#1293 | 27.97 | 2.52 | Positive |
| 97 | Sample#1294 | 40.98 | 504.99 | Positive |
| 98 | Sample#1302 | 29.52 | 2.96 | Positive |
| 99 | Sample#1308 | 81.48 | 3.02 | Positive |
| 100 | Sample#1313 | 19.74 | 3.69 | Positive |
| 101 | Sample#1323 | 31.71 | 2.81 | Positive |
| 102 | Sample#1329 | 38.24 | 1.55 | Positive |
| 103 | Sample#1330 | 25.65 | 2.67 | Positive |
| 104 | Sample#1331 | 31.19 | 1.30 | Positive |
| 105 | Sample#1336 | 64.10 | 1266.65 | Positive |
| 106 | Sample#1337 | 18.29 | 1.71 | Positive |
| 107 | Sample#1342 | 70.08 | 2.05 | Positive |
| 108 | Sample#1343 | 23.79 | 3.71 | Positive |
| 109 | Sample#1345 | 41.33 | 2.86 | Positive |
| 110 | Sample#1352 | 33.08 | 1.82 | Positive |
| 111 | Sample#1354 | 26.13 | 3.22 | Positive |
| 112 | Sample#1355 | 52.88 | 2.00 | Positive |
| 113 | Sample#1357 | 32.18 | 2.12 | Positive |
| 114 | Sample#1360 | 28.80 | 1.97 | Positive |
| 115 | Sample#1364 | 89.68 | 1.95 | Positive |
| 116 | Sample#1365 | 23.09 | 599.39 | Positive |
| 117 | Sample#1369 | 51.67 | 1.90 | Positive |
| 118 | Sample#1372 | 75.80 | 2.82 | Positive |
| 119 | Sample#1373 | 80.67 | 3.84 | Positive |
| 120 | Sample#1374 | 61.39 | 2.20 | Positive |
| 121 | Sample#1376 | 18.18 | 1.79 | Positive |
| 122 | Sample#1379 | 36.05 | 1.94 | Positive |
| 123 | Sample#1382 | 37.42 | 4.32 | Positive |
| 124 | Sample#1385 | 69.75 | 1174.82 | Positive |
| 125 | Sample#1389 | 47.71 | 1.79 | Positive |
| 126 | Sample#1390 | 18.12 | 3.18 | Positive |
| 127 | Sample#1392 | 26.92 | 2.15 | Positive |
| 128 | Sample#1400 | 19.79 | 6.75 | Positive |
| 129 | Sample#1401 | 41.08 | 2.07 | Positive |
| 130 | Sample#1405 | 51.63 | 2.12 | Positive |
| 131 | Sample#1406 | 47.13 | 1.08 | Positive |
| 132 | Sample#1410 | 42.77 | 1.99 | Positive |
| 133 | Sample#1416 | 28.91 | 2.09 | Positive |
| 134 | Sample#1424 | 93.33 | 1.12 | Positive |
| 135 | Sample#1432 | 34.38 | 1268.25 | Positive |
| 136 | Sample#1437 | 79.92 | 1.84 | Positive |
| 137 | Sample#1440 | 19.60 | 996.46 | Positive |
| 138 | Sample#1442 | 20.00 | 700.87 | Positive |
| 139 | Sample#1446 | 33.25 | 2.53 | Positive |
| 140 | Sample#1458 | 22.01 | 26.97 | Positive |
| 141 | Sample#1459 | 18.40 | 1.88 | Positive |
| 142 | Sample#1466 | 19.29 | 1.03 | Positive |
| 143 | Sample#1473 | 28.01 | 12.15 | Positive |
| 144 | Sample#1476 | 34.95 | 1.42 | Positive |
| 145 | Sample#1479 | 75.46 | 1.29 | Positive |
| 146 | Sample#1480 | 18.72 | 1.38 | Positive |
| 147 | Sample#1489 | 46.23 | 1.46 | Positive |
| 148 | Sample#1494 | 17.98 | 7.59 | Positive |
| 149 | Sample#1497 | 55.64 | 1.20 | Positive |
| 150 | Sample#1498 | 65.06 | 4.04 | Positive |
| 151 | Sample#1499 | 58.09 | 5.08 | Positive |
| 152 | Sample#1502 | 43.00 | 2.72 | Positive |
| 153 | Sample#1503 | 60.50 | 2.83 | Positive |
| 154 | Sample#1506 | 81.12 | 3.19 | Positive |
| 155 | Sample#1507 | 32.31 | 2.93 | Positive |
| 156 | Sample#1510 | 26.02 | 4.74 | Positive |
| 157 | Sample#1511 | 63.12 | 2.73 | Positive |
| 158 | Sample#1516 | 33.01 | 1.29 | Positive |
| 159 | Sample#1517 | 32.24 | 1321.97 | Positive |
| 160 | Sample#1518 | 53.13 | 1.10 | Positive |
| 161 | Sample#1519 | 35.34 | 3.88 | Positive |
| 162 | Sample#1520 | 25.89 | 837.99 | Positive |
| 163 | Sample#1521 | 19.18 | 2.01 | Positive |
| 164 | Sample#1522 | 44.50 | 798.71 | Positive |
| 165 | Sample#1531 | 26.25 | 4.23 | Positive |
| 166 | Sample#1540 | 19.50 | 2.42 | Positive |
| 167 | Sample#1545 | 36.55 | 805.95 | Positive |
| 168 | Sample#1547 | 74.21 | 2.14 | Positive |
| 169 | Sample#1554 | 18.74 | 19.21 | Positive |
| 170 | Sample#1555 | 21.16 | 12.63 | Positive |
| 171 | Sample#1558 | 32.50 | 2.11 | Positive |
| 172 | Sample#1561 | 25.81 | 1.09 | Positive |
| 173 | Sample#1591 | 18.34 | 2.75 | Positive |
| 174 | Sample#1595 | 27.21 | 1.00 | Positive |
| 175 | Sample#1597 | 31.94 | 1.46 | Positive |
| 176 | Sample#1600 | 76.81 | 1.21 | Positive |
| 177 | Sample#1606 | 34.85 | 5.40 | Positive |
| 178 | Sample#1612 | 18.49 | 2.35 | Positive |
| 179 | Sample#1613 | 68.96 | 3.54 | Positive |
| 180 | Sample#1618 | 21.46 | 2.13 | Positive |
| 181 | Sample#1620 | 59.33 | 3.53 | Positive |
| 182 | Sample#1621 | 19.16 | 1.67 | Positive |
| 183 | Sample#1625 | 20.65 | 768.65 | Positive |
| 184 | Sample#1628 | 59.54 | 8.75 | Positive |
| 185 | Sample#1629 | 49.77 | 3.88 | Positive |
| 186 | Sample#1646 | 44.58 | 8.93 | Positive |
| 187 | Sample#1647 | 57.33 | 2.01 | Positive |
| 188 | Sample#1648 | 34.82 | 18.55 | Positive |
| 189 | Sample#1663 | 22.23 | 1.96 | Positive |
| 190 | Sample#1664 | 22.60 | 2.40 | Positive |
| 191 | Sample#1666 | 25.02 | 1.31 | Positive |
| 192 | Sample#1669 | 53.54 | 1.56 | Positive |
| 193 | Sample#1686 | 53.66 | 2.24 | Positive |
| 194 | Sample#1688 | 44.39 | 1.85 | Positive |
| 195 | Sample#1690 | 25.83 | 2.00 | Positive |
| 196 | Sample#1694 | 23.37 | 13.50 | Positive |
| 197 | Sample#1696 | 55.02 | 2.08 | Positive |
| 198 | Sample#1700 | 61.85 | 2.42 | Positive |
| 199 | Sample#1713 | 18.96 | 16.36 | Positive |
| 200 | Sample#1715 | 18.90 | 1.52 | Positive |
| 201 | Sample#1716 | 43.26 | 3.63 | Positive |
| 202 | Sample#1724 | 34.02 | 19.46 | Positive |
| 203 | Sample#1732 | 21.41 | 1.94 | Positive |
| 204 | Sample#1738 | 21.61 | 1.50 | Positive |
| 205 | Sample#1749 | 20.07 | 9.73 | Positive |
| 206 | Sample#1750 | 18.36 | 4.40 | Positive |
| 207 | Sample#1762 | 52.70 | 2.20 | Positive |
| 208 | Sample#1770 | 70.78 | 1.07 | Positive |
| 209 | Sample#1783 | 18.25 | 354.49 | Positive |
| 210 | Sample#1796 | 25.59 | 1.16 | Positive |
| 211 | Sample#1804 | 31.28 | 5.46 | Positive |
| 212 | Sample#1806 | 18.84 | 2.76 | Positive |
| 213 | Sample#1809 | 28.20 | 1.72 | Positive |
| 214 | Sample#1816 | 47.99 | 3.61 | Positive |
| 215 | Sample#1831 | 33.93 | 6.50 | Positive |
| 216 | Sample#1886 | 29.80 | 1.17 | Positive |
| 217 | Sample#1895 | 23.66 | 9.49 | Positive |
| 218 | Sample#1930 | 20.62 | 2.25 | Positive |
| 219 | Sample#1933 | 45.69 | 209.11 | Positive |
| 220 | Sample#1947 | 27.66 | 194.92 | Positive |
| 221 | Sample#1953 | 32.00 | 15.09 | Positive |
| 222 | Sample#1956 | 35.14 | 1.98 | Positive |
| 223 | Sample#1961 | 34.66 | 151.90 | Positive |
| 224 | Sample#1968 | 21.74 | 180.72 | Positive |
| 225 | Sample#1970 | 44.20 | 3.84 | Positive |
| 226 | Sample#1971 | 20.65 | 2.91 | Positive |
| 227 | Sample#1975 | 19.93 | 1.24 | Positive |
| 228 | Sample#1976 | 30.56 | 2.01 | Positive |
| 229 | Sample#1989 | 75.01 | 1.47 | Positive |
| 230 | Sample#1990 | 17.86 | 15.70 | Positive |
| 231 | Sample#1992 | 24.70 | 67.69 | Positive |
| 232 | Sample#1997 | 66.80 | 1.93 | Positive |
| 233 | Sample#1998 | 38.39 | 28.48 | Positive |
| 234 | Sample#2005 | 45.69 | 18.46 | Positive |
| 235 | Sample#2007 | 37.93 | 2.31 | Positive |
| 236 | Sample#2013 | 21.62 | 4.68 | Positive |
| 237 | Sample#2014 | 42.95 | 5.62 | Positive |
| 238 | Sample#2017 | 21.39 | 4.68 | Positive |
| 239 | Sample#2019 | 27.55 | 1.28 | Positive |
| 240 | Sample#2031 | 63.24 | 1.38 | Positive |
| 241 | Sample#2086 | 24.24 | 62.17 | Positive |
| 242 | Sample#2088 | 26.27 | 5.44 | Positive |
| 243 | Sample#2092 | 27.81 | 2.72 | Positive |
| 244 | Sample#2093 | 49.14 | 251.85 | Positive |
