# Supplementary material for: Spatial and temporal clustering of anti-SARS-CoV-2 antibodies in Illinois household cats, 2021–2023
Source: PLoS One. 2024 May 2;19(5):e0299388. doi: 10.1371/journal.pone.0299388 (PMC11065222; doi:10.1371/journal.pone.0299388)
Supplement: S2 Table — (DOCX) [file pone.0299388.s002.docx]

**S2** **Table. ELISA, Virus-neutralizing assay, and Lumit™ Dx SARS-CoV-2 Immunoassay results for cat serum samples with inconsistent results.** bELISA cut off value = 17.6%; Lumit assay cut-off value = 1; VN, virus-neutralizing assay, “< 8” is interpreted as a negative result, in which no inhibition of SARS-CoV-2 infection/replication was detected at the lowest serum dilution (1:8).

| **Number** | **Sample ID** | **ELISA PI (%)** | **Lumit** | **VN (D614G)** | **VN (Omicron)** |
| --- | --- | --- | --- | --- | --- |
| 1 | Sample#200 | 22.85 | 0.01 | < 8 | < 8 |
| 2 | Sample#1347 | 30.57 | 0.69 | < 8 | < 8 |
| 3 | Sample#1366 | 23.6 | 0.74 | < 8 | < 8 |
| 4 | Sample#1383 | 32.09 | 0.72 | < 8 | < 8 |
| 5 | Sample#1422 | 21.15 | 0.03 | < 8 | < 8 |
| 6 | Sample#1431 | 18.13 | 0.03 | < 8 | < 8 |
| 7 | Sample#1433 | 35.4 | 0.79 | < 8 | < 8 |
| 8 | Sample#1493 | 62.71 | 0.03 | < 8 | < 8 |
| 9 | Sample#1803 | 19.73 | 0.48 | < 8 | < 8 |
