# Supplementary material for: Spatial and temporal clustering of anti-SARS-CoV-2 antibodies in Illinois household cats, 2021–2023
Source: PLoS One. 2024 May 2;19(5):e0299388. doi: 10.1371/journal.pone.0299388 (PMC11065222; doi:10.1371/journal.pone.0299388)
Supplement: Attachment — Submitted filename: Response to Reviewers.docx [file pone.0299388.s004.docx]

Responses to Journal requirements:

1. Please ensure that your manuscript meets PLOS ONE's style requirements, including those for file naming.

**Response:** The manuscript was re-formatted to meet PLOS ONE's style requirements

2. **Response:** The Funding Statement should be updated to: “This project was supported by the National Institute of Health [Grant #R01Al166791, to Y. Fang (PI) and D. Diel (MPI)].”
3. Please include your full ethics statement in the ‘Methods’ section.

**Response:** We included the following: ” Samples consisted of excess serum submitted for unrelated, clinical purposes to the laboratories. No blood was collected specifically for this study. Thus, the method of collection does not constitute animal use and did not require review by the University of Illinois Institutional Animal Care and Use Committee” (Lines 106-110).

4. We note that Figures 1, 3, 5, 6, and 7 in your submission contain [map/satellite] images which may be copyrighted.

**Response:** We used base maps provided by Arc GIS Pro and/or ArcGIS Online maps hosted by Esri. The appropriate attribution was provided at the bottom of each map.

Response to Reviewer 1’s comments.

**Reviewer #1:** In the manuscript, the authors described the spatio-temporal distribution of SARS-CoV-2 serological results in cats in Illinois. The study is interesting, as it combines geographical, spatial and biological analyses. Through these multidisciplinary approaches, the authors pointed some spatial and geographical occurences. It also clearly support the hypothesis of a SARS-CoV-2 transmission from human toward cat.

Introduction section

Line 69: Dogs can be also infected by SARS-CoV-2. Please add this animal species in your list and justify why in this study, the authors choose to focus only on cat infections.

**Response: “Dogs” was added to the list of animals that can be infected with SARS-CoV2 (lines 67 and 71). The following sentences and references were added to justify why this study focuses only on cat infections (lines 70-76).**

“SARS-CoV-2 has been reported to be transmitted from humans to animals, including domestic cats and dogs (15-18). Previous studies showed that SARS-CoV-2 did not replicate well in dogs, but was able to efficiently replicate in domestic cats and further transmit the virus to naive contact animals (19). A recent report provided evidence that domestic cats could transmit SARS-CoV-2 to humans (15). Since domestic household cats have close contact with humans, it is important to understand the prevalence of SARS-CoV-2 exposure in household cat populations.”

Result section

The cat sera were tested with different methodologies for the presence of SARS-CoV-2 antibody: ELISA, Luminex and seroneutralisation (D614G and Omicron). Please indicate the results for each methodologies in a supplemental table. Moreover, it would be intersting to add the titer of seroneutralisation for each virus tests. With these results, the authors could evaluate by which variant the cats have been infected. It would be an interesting supplementary information.

**Response: In our study, we initially screened all the cat samples by bELISA, and the bELISA positive samples were subsequently confirmed by Lumit assay. Due to the restrictions working in BSL3 lab, we only performed virus-neutralizing tests on those samples having inconsistent results between bELISA and Lumit assay.**

**To address this comment, we added Table S1 as a supplementary table to show the results of different antibody tests. We also clarified our antibody testing procedure in the Materials and Methods section (lines 118-133).**

Line 368: Specify whether you reported the human cases in Illinois or in United States in figure 8

**Response: We clarified that we referred to the human cases in Illinois.**

Discussion section

Lines 400-402: In this study, the authors found the higesht seropositivity in young cats. In the discussion, they should add the possibility that older cats may have been previously infected by SARS-CoV-2. They could also discuss the possiblity of cross-protection or not by previous feline coronavirus infection

**Response: We appreciate your excellent suggestion. The following sentences and reference were added in the discussion section (line 405-411):**

**“Another possibility is that some of the older cats may have been previously exposed/infected with SARS-CoV-2 or feline coronavirus (FCoV), and they could be protected from the SARS-CoV-2 infection by pre-existing immunity. A recent study demonstrated that both FCoV serotype 1 and 2 infected domestic cats developed cross-reactive antibodies against the SARS-CoV-2 receptor binding domain (40). Future studies are needed to elucidate the in-depth mechanisms that cause the difference in seroprevalence among various age groups of cats."**
